# Repeat breast-conserving treatment of ipsilateral breast cancer recurrence: a nationwide survey amongst breast surgeons and radiation oncologists in the Netherlands

**DOI:** 10.1007/s10549-021-06154-2

**Published:** 2021-03-13

**Authors:** Coco J. E. F. Walstra, Robert-Jan Schipper, Yvonne E. van Riet, Peter-Paul G. van der Toorn, Marjolein L. Smidt, Maurice J. C. vd Sangen, Adri C. Voogd, Grard A. P. Nieuwenhuijzen

**Affiliations:** 1 Department of Surgical Oncology, Catharina Ziekenhuis Eindhoven, Eindhoven, The Netherlands; 2 Department of Surgical Oncology Maastricht, Universitair Medisch Centrum, Maastricht, The Netherlands; 3 Department of Radiation Oncology, Catharina Ziekenhuis Eindhoven, Eindhoven, The Netherlands; 4 Department of Research, Netherlands Comprehensive Cancer Organisation (IKNL), Utrecht, The Netherlands; 5 GROW-School for Oncology and Developmental Biology, Maastricht University Medical Center, Maastricht, The Netherlands

**Keywords:** Survey, IBTR, Recurrent breast cancer, Repeat breast-conserving treatment, Re-irradiation

## Abstract

**Background:**

In line with the paradigm to minimize surgical morbidity in patients with primary breast cancer, there is increasing evidence for the safety of a repeat breast-conserving treatment (BCT) of an ipsilateral breast tumour recurrence (IBTR) in selected patients. The conditions for the feasibility of a repeat BCT vary widely in literature. In clinical practice, many physicians have ongoing concerns about the oncological safety and possible toxicity of repeat BCT.

**Aim:**

To investigate the attitude of Dutch breast surgeons and radiation oncologists towards repeat BCT and to report on their experiences with, objections against and perceived requirements to consider a repeat BCT in case of IBTR.

**Patients and methods:**

An online survey consisting of a maximum of 26 open and multiple-choice questions about repeat BCT for IBTR was distributed amongst Dutch breast surgeons and radiation oncologists.

**Results:**

Forty-nine surgeons representing 49% of Dutch hospitals and 20 radiation oncologists representing 70% of Dutch radiation oncology centres responded. A repeat BCT was considered feasible in selected cases by 28.7% of breast surgeons and 55% of radiation oncologists. The most important factors to consider a repeat BCT for both groups were the patient’s preference to preserve the breast and surgical feasibility of a second lumpectomy. Arguments against a repeat BCT were based on the perceived unacceptable toxicity and cosmesis of a second course of radiotherapy. The technique of preference for re-irradiation would be partial breast irradiation (PBI) according to all radiation oncologists. Differentiating between new primary tumours (NPT) and true recurrences (TR) was reported to be done by 57.1% of breast surgeons and 60% of radiation oncologists. The most important reason to differentiate between NPT and TR was to establish prognosis and to consider whether a repeat BCT would be feasible.

**Conclusion:**

An increasing number of Dutch breast cancer specialists is considering a repeat BCT feasible in selected cases, at the patient’s preference and with partial breast re-irradiation.

## Introduction

After breast-conserving treatment (BCT) for breast cancer, women remain at risk of an ipsilateral breast tumour recurrence (IBTR) of approximately 0.5% per year [1]. A salvage mastectomy has always been the surgical gold standard for the treatment of IBTR. However, the negative impact of a mastectomy compared to BCT on cosmetic outcome and quality of life (QoL) is well documented, at least after primary breast cancer treatment [2–6].

There is an ongoing paradigm to minimize morbidity caused by surgical interventions in patients with primary breast cancer, as is expressed by the increase in the use of breast-conserving surgery and the omission of axillary surgery in patients with a positive sentinel node [7, 8]. Similarly, interest has also grown to minimize surgical morbidity in patients with an IBTR without compromising the oncological outcome. The SNARB study showed that a repeat sentinel lymph node biopsy (rSLNB) in case of IBTR is oncologically safe regarding regional recurrences, regardless of the outcome of the rSLNB [9, 10]. It is even suggested to omit surgical nodal staging in patients with an IBTR, since the nodal status seems not to be an important prognostic factor [11]. In line with this paradigm, a repeat BCT would be the next step in minimizing surgical morbidity in patients with IBTR.

Several studies have investigated the feasibility of a repeat BCT for IBTR. A recent systematic review of 34 studies confirmed the oncological safety of a repeat BCT for selected cases of IBTR [12]. Nonetheless, the included studies were small and the patient series were highly selected. Selection criteria for eligibility for repeat BCT in the available literature are unifocality, small tumour size, a long recurrence-free interval, no evidence of nodal and/or distant metastases, a favourable tumour-to-breast ratio and, most importantly, the patient's preference to preserve the breast [12]. Oncological necessity of re-irradiation was confirmed with a 5-year local recurrence-free survival of 89% after repeat breast-conserving surgery (BCS) followed by radiotherapy versus 56% after repeat BCS alone. The 5-year overall survival was 77% after BCS alone versus 87% after BCS followed by re-irradiation (not statistically significant). The reported grade III–IV toxicity was low with a range from 2 to 21% and cosmesis was rated good to excellent in 75% of patients after repeat BCT with re-irradiation [12, 13].

Even though re-irradiation after repeat breast-conserving surgery seems to improve the oncological prognosis, the perceived high risks and potential toxicity of a second course of radiotherapy on the breast remain a barrier for acceptance. In daily practice, many radiation oncologists and surgeons continue to have concerns about the acute (wound healing problems, infection) and late (fibrosis, cosmesis, pain) toxicity of re-irradiation and state that this is the main reason why they would not consider a repeat BCT for IBTR. However, radiotherapy has evolved greatly over the years. Radiation techniques have been improved, optimizing the efficacy of the irradiation whilst minimizing damage to skin and surrounding organs [14, 15]. Also, the total dose has been reduced with less use of a boost. These developments have increased the feasibility of a second course of irradiation.

Over the years, the treatment of breast cancer has become a more and more multidisciplinary and dedicated approach. Breast surgeons, oncologists, radiation oncologists and radiologists work together to achieve a patient-tailored treatment plan. Within this team, breast surgeons and radiation oncologists are mostly involved with the challenges of repeat BCT. This study addresses the current attitude towards repeat BCT of both breast surgeons and radiation oncologists in the Netherlands. It provides insight in their experiences with, objections against and perceived requirements to consider a repeat BCT in case of IBTR, and also in current preoperative workup paradigms and surgical management of the axilla.

## Methods

### Participants

An online survey was sent out to all breast cancer providing hospitals and radiotherapy institutes in the Netherlands (a total of 68 and 20 centres, respectively) with the request to distribute it amongst their listed breast surgeons or radiation oncologists. The first invitation email contained the scope of the study and a link to the online survey. Reminders were sent out twice: the first after 6 weeks and the second after 12 weeks.

### Survey

The survey consisted of a maximum of 26 questions for the breast surgeons and 25 questions for the radiation oncologists, depending on which answers were filled out. The questions were either multiple choice or open. Both surveys are included in Appendix.

### Statistical analysis

Statistical analysis of the data obtained via through the surveys was performed using SPSS Statistics version 24. Most were descriptive analyses. Demographic differences between breast surgeons and radiation oncologists were compared using *X*-square test. To compare the use of diagnostic methods between respondents from university hospitals and community hospitals, a Chi-square test was used. A *p*-value < 0.05 was considered statistically significant.

To objectify and summarize the perceived importance of patient and tumour characteristics in differentiating between a 'true' recurrence or a new primary tumour (question 15 in both surveys), a weighted cumulative scoring system was used. Every score of 1 (most important) was awarded 6 points, counting down to 1 point to a score of 6 (least important). These points were then multiplied by the number of times; this score was chosen for that particular characteristic, resulting in a cumulative weighted score. This way, the highest cumulative score represents the highest importance awarded by the respondents.

## Results

### Surgeons

#### Participants

A total of 49 participants representing 33 (49%) of a total of 68 hospitals providing breast cancer care completed the survey. Demographics of the respondents are shown in Table 1.

**Table 1:** Demographics of respondents

	Breast surgeons (*N* = 49)	Radiation oncologists (*N* = 20)	*p*
Sex			
Male	26 (53.1%)	9 (45.0%)	0.884
Female	23 (46.9%)	11 (55.0%)	
Age			
< 40	7 (14.3%)	5 (25.0%)	0.449
41–50	22 (44.9%)	5 (25.0%)	
51–60	16 (32.7%)	8 (40.0%	
> 61	4 (8.2%)	2 (10.0%)	
Years of experience			
< 5	5 (10.2%)	6 (30.0%)	0.097
5–10	10 (20.4%)	2 (10.0%)	
10–15	13 (26.5%)	2 (10.0%)	
> 15	21 (42.9%)	10 (50.0%)	
Time spent on breast cancer patients			
< 25%	5 (10.2%)	3 (15.0%)	0.918
25–50%	17 (34.7%)	6 (30.0%)	
50–75%	14 (28.6%)	5 (25.0%)	
> 75%	13 (26.5%)	6 (30.0%)	
Working in			
University hospital	9 (18.4%)	8 (40.0%)	0.051
Large non-university hospital	34 (69.4%)	9 (45.0%)	
Community hospital	6 (12.2%)	3 (15.0%)	

#### Surgical treatment experiences and arguments

Ninety-eight percent of all surgeons report that an IBTR is treated in their hospital. According to 71.4% of the responders, a salvage mastectomy is the gold standard in case of IBTR, whereas 28.6% consider a repeat BCT to be feasible in some cases. Forty-nine percent of the surgeons report to have experience with repeat BCT for IBTR in their practice. In all cases, this was outside the context of a clinical study. Table 2 shows the breast surgeons’ responses to questions on their experience with and attitude towards repeat BCT.

**Table 2:** Experience with and attitude towards repeat BCT for IBTR

	Breast surgeons (*N* = 49)	Radiation oncologists (*N* = 20)
Reasons for repeat BCT for IBTR in the past	*Answered by 23 (46.9%)*	*Answered by 10 (50.0%)*
Patient’s preference	13 (56.5%)	5 (50.0%)
Small, low-grade tumour	8 (34.8%)	3 (33.3%)
High patient age	6 (26.1%)	3 (33.3%)
Omittance of radiotherapy after primary BCT	6 (26.1%)	2 (20.0%)
Favourable tumour-to-breast ratio	5 (21.7%)	3 (33.3)
New primary tumour	2 (8.7%)	2 (20.0%)
Arguments against repeat BCT for IBTR	*Answered by 49 (100%)*	*Answered by 10 (50.0%)*
Need for re-irradiation	39 (79.6%)	7 (70.0%)
Higher risk of local re-recurrence	25 (51.0%)	4 (40.0%)
No acceptable cosmesis after repeat BCT	22 (44.9%)	6 (60.0%)
High risk of wound healing problems	18 (36.7%)	4 (40.0%)
High risk of irradical margins	3 (6.1%)	2 (20.0%)
Feasible conditions to consider repeat BCT for IBTR	*Answered by 49 (100%)*	*Answered by 10 (50.0%)*
Patient’s preference to preserve the breast	42 (85.7%)	9 (90.0%)
Favourable tumour-to-breast ratio	41 (83.7%)	7 (70.0%)
Unifocal tumour	35 (71.4%)	7 (70.0%)
Opportunities for oncoplastic reconstruction	29 (59.2%)	2 (20.0%)
Opportunities for partial breast re-irradiation	26 (53.1%)	8 (80.0%)
Omittance of radiotherapy after primary BCT	24 (49.0%)	6 (60.0%)
New primary tumour	14 (28.6%)	–

#### Differentiating between new primary tumour and 'true' recurrence

Slightly more than half of the respondents (57.1%) reported to differentiate between a new primary tumour (NP) and a 'true' recurrence (TR), of which 39.3% use clonality testing to differentiate. This was not significantly different between surgeons working in university hospitals/specialized oncology clinics and non-university or community hospitals (*p* = 0.166). Arguments pro and against differentiating between TR and NPT are listed in Table 3.

**Table 3:** Arguments to differentiate between TR (true recurrences) and NPT (new primary tumours)

	Breast surgeons (*N* = 49)	Radiation oncologists (*N* = 20)
Arguments *pro* differentiating TR from NPT	*Answered by 21 (42.9%)*	*Answered by 12 (60.0%)*
Prognosis (assumingly worse for TR)	16 (76.2%)	6 (50.0%)
An NPT would render a repeat BCT feasible	5 (23.8%)	6 (50.0%)
Arguments *contra* differentiating TR from NPT	*Answered by 19 (38.8%)*	*Answered by 8 (40.0%)*
No influence on therapy decision making	8 (42.1%)	6 (75.0%)
A second course of radiotherapy is undesirable	7 (36.8%)	–
No reliable distinction methods	4 (21.1%)	2 (25.0%)

When asked for factors influencing the perceived probability of a true recurrence, the surgeons selected the following tumour characteristics in order of importance:

1. Within or adjacent to the primary lumpectomy scar (cumulative score of 245 points)
2. Same quadrant of the breast as primary tumour (cumulative score of 181 points)
3. Receptor status (HER2, progesterone and estrogen expression) (cumulative score of 170 points)
4. Similar tumour subtype (e.g. ductal, lobular carcinoma) as primary tumour (cumulative score of 169 points)
5. Time to recurrence (cumulative score of 162 points)
6. Clonality comparison (cumulative score of 157 points)
  Two surgeons added the options:
7. Within the biopsy track of the primary tumour
8. Age

#### Dissemination workup

In case of IBTR, 26.5% of the surgeons always perform a full staging preoperative workup to assess for regional and distant metastasis, whereas 59.2% only performed this in case of a 'true' recurrence, and 10% only in case of clinical suspicion of distant metastasis. The majority of surgeons (91.2%) prefer PET-CT for this workup and 9.8% use a CT scan of the chest and abdomen and a bone scintigraphy. A standard ultrasound (US) of the ipsilateral axilla is performed by 87.8% of surgeons, whereas 6% do this always bilaterally, 4% only perform an axillary US in case of palpable lymph nodes and 2% only after positive lymph nodes on a PET-CT scan.

#### Surgical treatment of the axilla

The majority of surgeons (85.7%) prefer to perform an rSLNB first. Most surgeons choose to omit an ALND in case of a negative rSLNB (91.8%), and 51% do the same in case of a positive rSLNB. Seven surgeons (14.3%) perform an ALND without a rSLNB, of which six (12.2%) after a positive cytologic biopsy of the axilla (cN+) and one (2%) in all patients with IBTR. Nine surgeons (18.4%) rely on a negative cytologic biopsy to omit both rSLNB and ALND.

When an ALND has already been performed during surgery for the primary tumour, 65.3% of surgeons omit a repeat ALND.

### Radiation oncologists

#### Participants

Twenty radiation oncologists representing 14 (70%) of a total of 20 breast cancer radiotherapy institutes filled out the survey. Demographics of the respondents are shown in Table 1.

#### Re-irradiation in repeat BCT

An IBTR was treated in 90% of the respondents' hospitals (at least surgically). Forty-five percent of the radiation oncologists regards a salvage mastectomy as the gold standard. The others (55%) agree that in some cases a repeat BCT could be feasible.

Half of the respondents (50%) reported to have experience with (re)irradiation of an IBTR. Of these, 30% only had experience with whole-breast re-irradiation and 70% also with partial breast re-irradiation after repeat lumpectomy.

Table 2 shows the responses of the radiation oncologists on their attitude towards repeat BCT.

Twenty percent of the radiation oncologists remain sceptical towards a second course of radiotherapy and would probably never consider it. About 75% of the respondents would be prepared to consider re-irradiation, under selected circumstances and with more available evidence for its safety and feasibility.

#### Technique preferences

All participants considering re-irradiation for IBTR prefer PBI above WBI. Ten percent would rather apply brachytherapy, 10% intraoperative radiotherapy (IORT) and 25% external beam PBI. All others did not specify a preferred technique for PBI.

#### Differentiating between new primary tumour and 'true' recurrence

Sixty percent of the respondents differentiate between NP and TR (see Table 3). The following factors are regarded important in differentiating between NP and TR, in order of importance:

1. Clonality analysis (used by 36.4% of radiation oncologists differentiating between NP and TR) (cumulative score of 95 points)
2. IBTR in scar of primary lumpectomy (cumulative score of 85 points)
3. Time to recurrence (shorter is more prone to be a TR) (cumulative score of 79 points)
4. Receptor status identical to primary tumour (cumulative score of 67 points)
5. Identical tumour type (IDC/ILC) as primary tumour (cumulative score of 66 points)
6. IBTR in same quadrant as primary tumour (cumulative score of 61 points)

### Breast surgeons vs. radiation oncologists

Demographics and group composition between breast surgeons and radiation oncologists did not differ significantly (see Table 1). When asked whether they would consider a repeat BCT when feasible, 28.9% of breast surgeons and 55% of radiation oncologists replied in a positive way (*p* = 0.008).

## Discussion

Growing evidence suggests that repeat BCT is a safe and feasible option in selected patients with IBTR, and re-irradiation of the breast seems to improve prognosis [12]. However, restraint towards this principle persists amongst treating physicians, assumingly for oncological and toxicity reasons. This study addressed the current attitude of breast surgeons and radiation oncologists in the Netherlands. With a coverage of 49% of the Dutch surgical breast cancer centres and 70% of the radiotherapy institutions, this study offers a representative impression of the current practices and opinions in the Netherlands.

The patient's preference to preserve the breast appears the most important reason for both groups to consider a repeat BCT. This is in line with preceding studies on the feasibility of repeat BCT in IBTR, always using this as a selection criterion. Besides this crucial argument in favour of repeat BCT, breast surgeons tend to focus on the surgical workability of the tumour (unifocality, a favourable tumour-to-breast ratio, cosmetic outcome), providing the oncological safety is acceptable. Radiation oncologists are more concerned about toxicity of a second course of radiotherapy, but are willing to consider re-irradiation under the right circumstances and with more evidence on the safety and feasibility. According to this survey, a unifocal, N0 tumour with a favourable tumour-to-breast ratio in a patient with a wish to preserve her breast could be considered eligible for repeat BCT, providing partial breast re-irradiation techniques are available.

This survey showed a markedly higher willingness towards repeat BCT for radiation oncologists compared to breast surgeons. This could not be explained by any demographic differences between the groups. However, a possible explanation could be that radiation oncologists have more experience with partial breast re-irradiation, which is appointed an important condition to consider repeat BCT (for both breast surgeons and radiation oncologists in this survey).

Both breast surgeons and radiation oncologists feared a higher risk to develop a second local recurrence after repeat BCT in comparison to mastectomy, but did not prioritize this in their objections against BCT (the breast surgeons put this argument second and the radiation oncologists last). A recent systematic review showed a 5-year local control of 89% after repeat BCS followed by re-irradiation. The RTOG-1014 group recently published follow-up data from their prospective trial with 3D-conformal partial breast irradiation and showed only 5% local recurrence after 5 years [16]. In comparison, the SNARB study in which 94.6% of patients underwent salvage mastectomy after IBRT showed a 5-year local control of 96.1% [11]. These percentages show that repeat BCT provides a low probability of local re-recurrence and seems to be comparable to patients treated with a salvage mastectomy.

To optimize the oncological safety of a repeat BCT, adequate patient selection is of vital importance. This includes reliable imaging of the IBTR to assess multifocality and size. A recent study in mastectomy specimens showed a higher multifocality rate in IBTR than in primary breast cancer [17]. Most of the series describing a repeat BCT did not specify the use of breast MRI in preoperative assessment. A recent study showed that MRI is superior to XMG and/or US in detecting multifocality and assessing tumour size in IBTR [18]. The use of MRI in the workup of patients eligible for a repeat BCT would therefore result in a more adequate selection of patients.

To date, very little literature exists discussing the difference between the short-term morbidity of salvage mastectomy versus repeat lumpectomy [19]. The surgical complication rate after primary mastectomy (varying from seroma, wound infection and wound dehiscence) varies between 10 and 30% in recent studies [20, 21]. The incidence of seroma and other short-term complications in patients with salvage mastectomy after previous irradiation as part of a primary breast-conserving treatment varies from 17 to 27% [22–24]. One would expect that repeat breast-conserving surgery in a previously irradiated area could yield wound healing problems, leading to delay of the re-irradiation, a worse cosmetic outcome or ultimately the need for a salvage mastectomy. The limited evidence on this important topic is promising and long-term complications and cosmesis seem acceptable [25–27], but generally, this outcome is not commented upon. It would be a valuable addition to the many studies currently investigating repeat BCT to report on these outcomes, to be able to compare postoperative wound healing problems with those in salvage mastectomy patients.

The evidence for the relatively low toxicity of a repeated course of radiotherapy in IBTR is rapidly increasing. In literature, a clear preference appears for partial breast irradiation after repeat lumpectomy [12]. Various techniques for PBI are external beam partial radiotherapy, brachytherapy and intraoperative radiotherapy (IORT). The oncological safety of all three techniques seems promising with high patient satisfaction [12, 19]. Intraoperative radiotherapy would offer the advantage of a single fraction, resulting in patient convenience, as opposed to other partial breast irradiation modalities which require various hospital visits. Furthermore, it offers the opportunity for immediate oncoplastic reconstruction of the breast, as the replaced tissue will not be irradiated postoperatively.

### New primary tumour vs. true recurrence

When asked about the role of the nature of the IBTR (i.e. true recurrence or new primary tumour) in considering a repeat BCT, 57% of surgeons and 60% of radiation oncologists would take this into account. Their experiences and opinions on whether to and how to differentiate between TR and NP vary. Tumour type, receptor status, location of the IBTR near the primary tumour and time to recurrence are all characteristics that have been described before in studies on repeat BCT [28, 29]. Evidence for the accuracy of these methods remains unclear, but the outcomes for assumed TRs seem worse than for NPs [29, 30]. Clonality comparison is trusted by more than 30% of both breast surgeons and radiation oncologists in this survey. Previous studies on repeat BCT differentiating between TR and NP did not include clonality analysis.

### Dissemination workup

As expected, most breast surgeons perform an (at least ipsilateral) axillary ultrasound in case of IBTR. The number of breast surgeons performing a full workup for distant metastases in all cases of IBTR is limited. Most surgeons rely on tumour characteristics and other methods to define the nature of the IBTR (i.e. TR or NP) and physical examination to decide whether extensive imaging is indicated. PET-CT is their modality of choice.

As to surgical management of regional metastases, the majority of surgeons currently perform an rSLNB instead of an ALND in clinically node negative patients with an IBTR. However, the prognostic significance of an rSLNB seems limited; the SNARB study showed that regional and distant metastasis-free survival did not significantly differ after positive, negative or unsuccessful [11, 31].

### Limitations and strengths

Due to the voluntary nature of this survey, a selection bias cannot be ruled out. Responders having affinity with repeat BCT could have been more prone to fill out the survey. A response bias is inevitable with survey-based results. We tried to avoid this effect as much as possible by leaving always one 'open' option at the end of multiple-choice questions.

This survey was, to our knowledge, the first to assess the current attitude towards repeat BCT amongst breast cancer specialists dealing with IBTR. The broad coverage of breast cancer centres yields a representative and clarifying impression of the current arguments in favour and against repeat BCT.

## Conclusion

This survey showed that, using strict selection criteria and at the patient's preference, more than half of the Dutch breast cancer specialists consider a repeat BCT for IBTR. They urge for more evidence on the safety and feasibility of this treatment. Current literature on this subject is growing rapidly. More evidence on the technique of preference for re-irradiation is needed.

## Declarations

### Research involving human and/or animal participants

This article does not contain any studies with human participants or animals performed by any of the authors.
